# Advances in auxin synthesis, transport, and signaling in rice: implications for stress resilience and crop improvement

**DOI:** 10.3389/fpls.2024.1516884

**Published:** 2025-01-20

**Authors:** Mengmeng Hou, Yuanbo Zhang, Xinyi Xu, Hao Ai

**Affiliations:** ^1^ Guangdong Laboratory of Lingnan Modern Agriculture, Genome Analysis Laboratory of the Ministry of Agriculture and Rural Affairs, Agricultural Genomics Institute at Shenzhen, Chinese Academy of Agricultural Sciences, Shenzhen, China; ^2^ State Key Laboratory of Crop Stress Adaptation and Improvement, School of Life Sciences, Henan University, Kaifeng, China; ^3^ College of Plant Science and Technology, Huazhong Agricultural University, Wuhan, Hubei, China; ^4^ Center for Crop Biotechnology, College of Agriculture, Anhui Science and Technology University, Fengyang, China

**Keywords:** auxin biosynthesis, transport, signal transduction, metabolism, rice

## Abstract

Auxin, a crucial plant hormone, plays a pivotal role in regulating various aspects of rice growth and development, including cell elongation, root formation, and responses to environmental stimuli. Recent breakthroughs in auxin research have revealed novel regulatory mechanisms, such as the identification of auxin-related genes like DNR1 and OsARF18, which enhance rice nitrogen use efficience and resistance to glufosinate. Additionally, advancements in understanding auxin transport and signaling pathways have highlighted their potential in optimizing tillering, root architecture, and grain yield. This review examines these molecular mechanisms and their interactions with other hormones, emphasizing their integration into breeding programs for improved rice productivity. By synthesizing these findings, we provide a comprehensive overview of how auxin research informs strategies for developing rice varieties with enhanced adaptability and optimized growth, contributing to food security and sustainable agriculture.

## Introduction

Auxin is a crucial phytohormone that regulates various aspects of plant growth and development (Robert et al., 2015; Shuai et al., 2016), playing an essential role in shaping the architecture of plants. In rice (Oryza sativa), a staple crop of global importance, auxin influences a wide range of physiological processes, including cell elongation (Wang et al., 2019; Ma and Li, 2019), root and shoot development (Chen et al., 2012; Hou et al., 2021), vascular differentiation (Wang et al., 2020), and responses to environmental stimuli (Wang et al., 2019, 2023). Understanding the complex mechanisms of auxin signaling and its regulatory networks in rice has been a focal point of plant science research.

Rice production faces significant challenges, including increasing global food demand driven by population growth, the adverse impacts of climate change such as drought and salinity, and the need to reduce agricultural inputs while maintaining high yields. These challenges necessitate the development of rice varieties with improved stress resilience, enhanced nutrient use efficiency, and optimized growth characteristics. Auxin, as a key regulator of plant development and environmental responses, offers promising avenues for addressing these issues through targeted genetic and agronomic interventions.

Recent advances in molecular biology and genomics have provided deeper insights into how auxin modulates rice growth, offering potential strategies for improving crop yield and stress tolerance. This review systematically summarizes the latest progress in auxin research in rice, highlighting the hormone’s diverse roles in plant development and the underlying molecular mechanisms, with a focus on its application in overcoming current agricultural challenges.

## Auxin synthesis and its regulation

**Figure 1:**
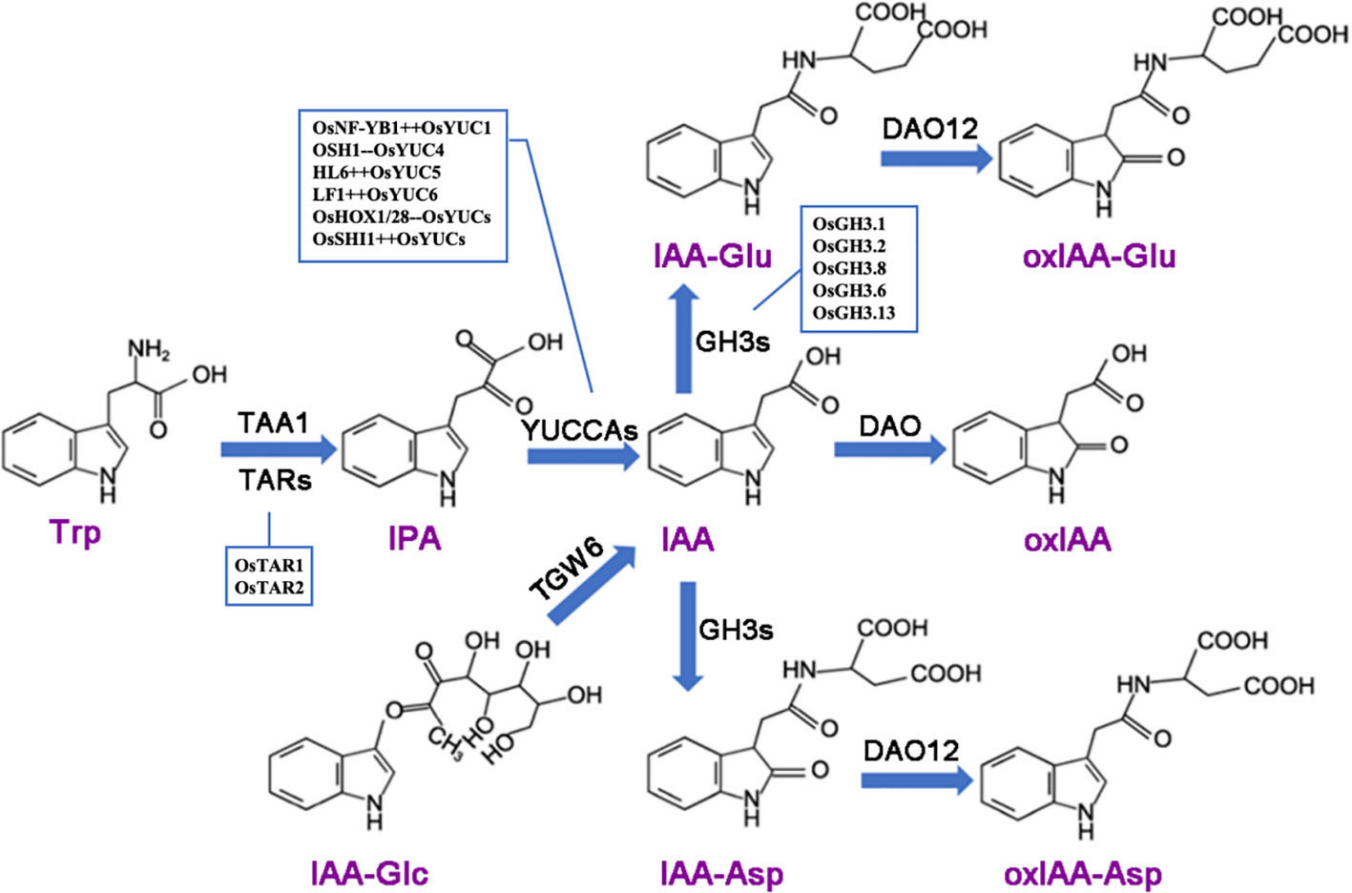
Auxin metabolism involves several key enzymatic processes. The TAA (tryptophan aminotransferase) family catalyzes the conversion of tryptophan (Trp) into indole-3-pyruvic acid (IPA), which is subsequently converted into indole-3-acetic acid (IAA) by the YUCCA (YUC) family. The enzyme TGW6 hydrolyzes IAA-glucose to release free IAA. IAA can be conjugated with amino acids through the action of GH3 (IAA-amide synthetase) or metabolized by DAO (dioxygenase for auxin oxidation) into 2-oxoindole-3-acetic acid (oxIAA). Additionally, *in vitro* studies have shown that DAO12 can oxidize IAA-aspartate (IAA-Asp) and IAA-glutamate (IAA-Glu) into 2-oxoindole-3-acetic acid-aspartate (oxIAA-Asp) and 2-oxoindole-3-acetic acid-glutamate (oxIAA-Glu), respectively. The plus sign (++) following a gene name indicates positive regulation, while the minus sign (–) denotes negative regulation.

Plants have evolved a complex network to regulate auxin concentration, ensuring optimal growth. Auxin levels in plant cells are controlled through biosynthesis, conjugation with other molecules, and irreversible degradation (**Figure 1**). In rice, the TAA (Tryptophan aminotransferase of Arabidopsis)/YUCCA (YUC)-mediated two-step pathway is the primary auxin biosynthesis route. Disruption of the YUC or TAA genes leads to pleiotropic phenotypes and a reduction in auxin levels (Yoshikawa et al., 2014; Wang Y. et al., 2018). YUCCA is also a rate-limiting enzyme in auxin biosynthesis. Auxin synthesized via the TAA/YUCCA pathway is critical for rice development. For example, auxin activates the transcription of OsWOX11 (WUSCHEL-RELATED HOMEOBOX11), driving the initiation and development of crown roots, establishing the YUCCA-Auxin-WOX11 module for rice crown root development (Zhang et al., 2018). The OsFIB (FISH BONE)/OsTAA1 gene, encoding a tryptophan aminotransferase, plays a key role in auxin synthesis. The loss of function in OsFIB/OsTAA1 leads to various abnormal phenotypes, including smaller leaves, larger leaf angles, abnormal vascular tissue development, spikelet and root defects, reduced IAA levels, and defective polar auxin transport (Yoshikawa et al., 2014). *OsYUC11* is a key factor in auxin biosynthesis in rice endosperm and is a dynamically imprinted gene. It predominantly expresses the paternal allele in the endosperm 10 days after fertilization and becomes non-imprinted 15 days after fertilization. The functional maternal allele of OsYUC11 can restore the paternal defect of the gene (Xu et al., 2021). Knockout of OsYUC11 hinders grain filling and storage product accumulation, which can be restored by exogenous auxin application. The transcription factor OsNF-YB1 (Nuclear Factor YB1) directly interacts with the OsYUC11 promoter, activating its expression and promoting auxin biosynthesis. The increased auxin levels stimulate the expression of genes related to sucrose metabolism and starch synthesis in endosperm cells, thereby increasing starch accumulation and grain size (Xu et al., 2021; Zhang et al., 2021a).

Root hairs enhance root penetration into compacted layers by elongating in response to elevated auxin levels during root compaction. This response is mediated by the auxin synthesis gene OsYUCCA8, expressed in the root apex and induced by compaction stress. The auxin influx carrier OsAUX1 transports the auxin signal from the root apex to the root hair zone, promoting elongation. Mutants of OsYUCCA8, OsAUX1, or root-hair-specific genes exhibit shorter root hairs and reduced penetration ability, underscoring the importance of this auxin-mediated mechanism in root adaptation to compacted soil (Kong et al., 2024). During the ethylene response in rice roots, the ethylene signaling pathway promotes the accumulation of OsEIL1 and stimulates auxin biosynthesis. The ethylene-induced accumulation of xylose residues and xyloglucan (XyG) depends on the function of OsTAR2, a tryptophan aminotransferase involved in the auxin biosynthesis pathway. OsEIL1 works in concert with the auxin pathway to upregulate the expression of OsCSLC1, OsCSLC2, OsCSLC7, and OsCSLC9 in the root tip. This upregulation leads to XyG accumulation, which restricts the loosening of cellulose microfibrils in the epidermal cell walls of the root elongation and differentiation zones.

Phylogenetic analysis identified OsDNR1 (DULL NITROGEN RESPONSE1) as homologous to AtVAS1 (REVERSAL OF SAV3 PHENOTYPE 1), an aminotransferase involved in converting indole-3-pyruvate to L-tryptophan, thus antagonizing auxin biosynthesis. A 520 bp variation in the OsDNR1 promoter between indica and japonica rice affects expression levels, with lower expression in indica leading to higher auxin content and enhanced nitrogen uptake. Knockout of *OsDNR1* in japonica rice increases yield by 8%-25% under varying nitrogen conditions, highlighting its potential for improving nitrogen use efficiency (Zhang et al., 2021c).

Several upstream genes affect rice growth and stress response by regulating auxin synthesis. OsFTIP7 (FT-INTERACTING PROTEIN 7) can interact with OSH1/Oskn1 (KNOX family class 1 homeobox gene) and regulate the cytoplasmic-nuclear shuttle of OSH1. After entering the nucleus, OSH1 can bind to the promoter region of OsYUCCA4, a key gene for auxin synthesis, directly inhibiting OsYUCCA4 transcription, thereby downregulating auxin levels, promoting anther dehiscence, and ultimately controlling rice fertility (Song et al., 2018). OsFTIP7 also participates in metal oxide nanoparticle (NPs) mediated physiological and biochemical changes through negative regulation of auxin biosynthesis in rice. The loss of function of OsFTIP7 reduces the toxicity of CuO and ZnO to seedlings by accumulating more biomass and chlorophyll content. High exposure to metal oxide NPs results in higher auxin levels in *osftip7*, caused by increased expression of auxin biosynthesis genes. Additionally, IAA-treated seedlings show phenotypes similar to osftip7 under high concentrations of CuO and ZnO (Jiang et al., 2021). OsHOX1 (homeodomain-leucine zipper gene) and OsHOX28 bind to the HSFA2D (HEAT STRESS TRANSCRIPTION FACTOR 2D) promoter to inhibit its expression. Compared with HSFA2D and LA1, OsHOX1 and OsHOX28 attenuate auxin lateral transport, inhibiting the expression of WUSCHEL-related homeobox genes WOX6 and WOX11 under gravity. Furthermore, OsHOX1 and OsHOX28 inhibit the expression of multiple OsYUCCAs, reducing auxin synthesis. Therefore, OsHOX1 and OsHOX28 regulate local auxin distribution by inhibiting the HSFA2D-LA1 pathway and reducing endogenous auxin content, thus controlling rice tillering angle (Hu et al., 2020). OsWOX3B (WUSCHEL-like homeobox gene), which encodes a protein with a homologous domain (HD), is crucial for epidermal hair initiation. Yeast two-hybrid and bimolecular fluorescence complementation assays reveal that HL6 (hairy leaf 6) interacts with OsWOX3B’s HD domain to form heterodimers, as well as homodimers. OsWOX3B enhances HL6’s binding to OsYUCCA5 and positively regulates HL6 expression at the transcriptional level, co-regulating auxin-related genes during epidermal hair formation (Sun et al., 2017). OsWOX3B also plays a role in establishing leaf bilateral symmetry (Honda et al., 2018).

The gene RNR10 (REGULATOR OF N-RESPONSIVE RSA ON CHROMOSOME 10) is responsible for the significant differences in root developmental plasticity in response to nitrogen levels between the indica (Xian) and japonica (Geng) subspecies of rice. RNR10 encodes an F-box protein that interacts with DNR1 (DULL NITROGEN RESPONSE1), a negative regulator of auxin biosynthesis. Through monoubiquitination, RNR10 inhibits the degradation of DNR1, thereby antagonizing auxin accumulation, which results in reduced root responsiveness to nitrogen and nitrate (NO^3-^) uptake (Huang et al., 2023). Additionally, ASL (argininosuccinate lyase) mitigates ammonium-inhibited root elongation by converting excess glutamine to arginine, leading to auxin accumulation in the root meristem and stimulating elongation. Natural variation in the ASL allele between japonica and indica subspecies accounts for their differing root sensitivity to ammonium (Xie et al., 2023). The *shi1* (short internodes 1) mutant exhibits auxin-deficient root development, brassinosteroid (BR)-deficient architecture and grain size, and enhanced ABA-mediated drought tolerance. This mutant is hyposensitive to auxin and BR but hypersensitive to abscisic acid (ABA). OsSHI1 promotes auxin and BR biosynthesis by activating OsYUCCAs and D11(dwarf 11) expression while suppressing ABA signaling through the induction of OsNAC2 (NAC transcription factor), an ABA signaling repressor. Furthermore, transcription factors OsARF19, LIC, OsZIP26, and OsZIP86 directly bind to the OsSHI1 promoter, regulating its expression in response to auxin, BR, and ABA (Duan et al., 2023). These insights suggest potential applications in crop improvement by targeting specific genes to enhance stress tolerance and nutrient efficiency in rice.

## Auxin transport

### AUX1/LAX family

The AUX1/LAX gene family in rice comprises five members involved in auxin transport. OsAUX1, OsAUX3, and OsAUX4 have been extensively studied. OsAUX1 is ubiquitously expressed but shows high expression in the stele, root tip, lateral root primordia, and lateral roots. Its expression can be induced by IAA, NAA, 6-BA, and cadmium (Yu et al., 2015; Zhao et al., 2015). OsAUX1 negatively regulates primary root length but positively influences root hair development and cadmium stress response (Yu et al., 2015). It plays a role in auxin polar transport and lateral root initiation in rice (Zhao et al., 2015). Transcription factors and epigenetic modifications also affect OsAUX1 expression. For example, OsWOX4, a WUSCHEL-related homeobox gene, activates OsAUX1 to regulate primary root elongation (Chen et al., 2020a). *OsRLR4* (root length regulator 4), a PRAF subfamily member, interacts with OsTrx1 (thioredoxin) to promote H3K4me3 deposition on the OsAUX1 promoter, altering its transcriptional activity (Sun et al., 2022). In osaux1 mutants, root angle changes promote phosphorus foraging in topsoil, although phosphorus uptake efficiency remains unchanged. Under low phosphorus conditions, OsAUX1 aids in moving auxin from the root tip to the differentiation zone, promoting root hair elongation (Giri et al., 2018). Thus, OsAUX1 is crucial for phosphorus foraging in rice.

OsAUX3 functions as an auxin influx transporter, essential for apical auxin transport. It positively regulates primary root elongation by maintaining meristem cell division and may influence lateral root initiation through cell cycle-related genes, though it negatively affects root hair elongation (Wang et al., 2019). OsAUX3 also plays a role in aluminum stress response; the *osaux3-2* mutant shows reduced sensitivity to aluminum, lower auxin concentration, and less oxidative damage compared to wild-type (Wang et al., 2019). The *miR167a-OsARF6-OsAUX3* module regulates grain length and weight, with miR167a positively influencing these traits by silencing OsARF6, which binds to the OsAUX3 promoter (Qiao et al., 2021). OsAUX4 is highly expressed in primary, lateral, and adventitious roots, including root hairs. The *osaux4* mutant exhibits a shorter primary root but longer root hairs, indicating OsAUX4’s role in promoting primary root elongation while inhibiting root hair elongation. OsAUX4 also affects phosphorus starvation responses by altering the expression of phosphorus-related genes, linking auxin and phosphorus signaling (Ye et al., 2021).

### PIN family

The PIN family of auxin efflux transporters is crucial for auxin distribution between cells. OsPIN1 includes four homologs (OsPIN1a - OsPIN1d), with OsPIN1b functioning as an auxin efflux transporter involved in adventitious root and tiller development (Xu et al., 2005). The pin1apin1b double mutant shows reduced primary root and bud lengths, fewer crown roots, altered root orientation, and increased root hair length. The pin1cpin1d double mutant displays a naked spikelet phenotype at flowering (Li et al., 2019b; 2022b), indicating functional diversity among OsPIN1 paralogs. OsPIN1b also responds to nitric oxide and strigolactone, promoting root tip meristem activity and primary root elongation under low nitrogen and phosphorus conditions (Sun et al., 2018). OsPIN1b and PILS6b are involved in tillering development, with OsSPL14 activating their expression, negatively correlating with axillary bud development (Li et al., 2022a).

**Figure 2:**
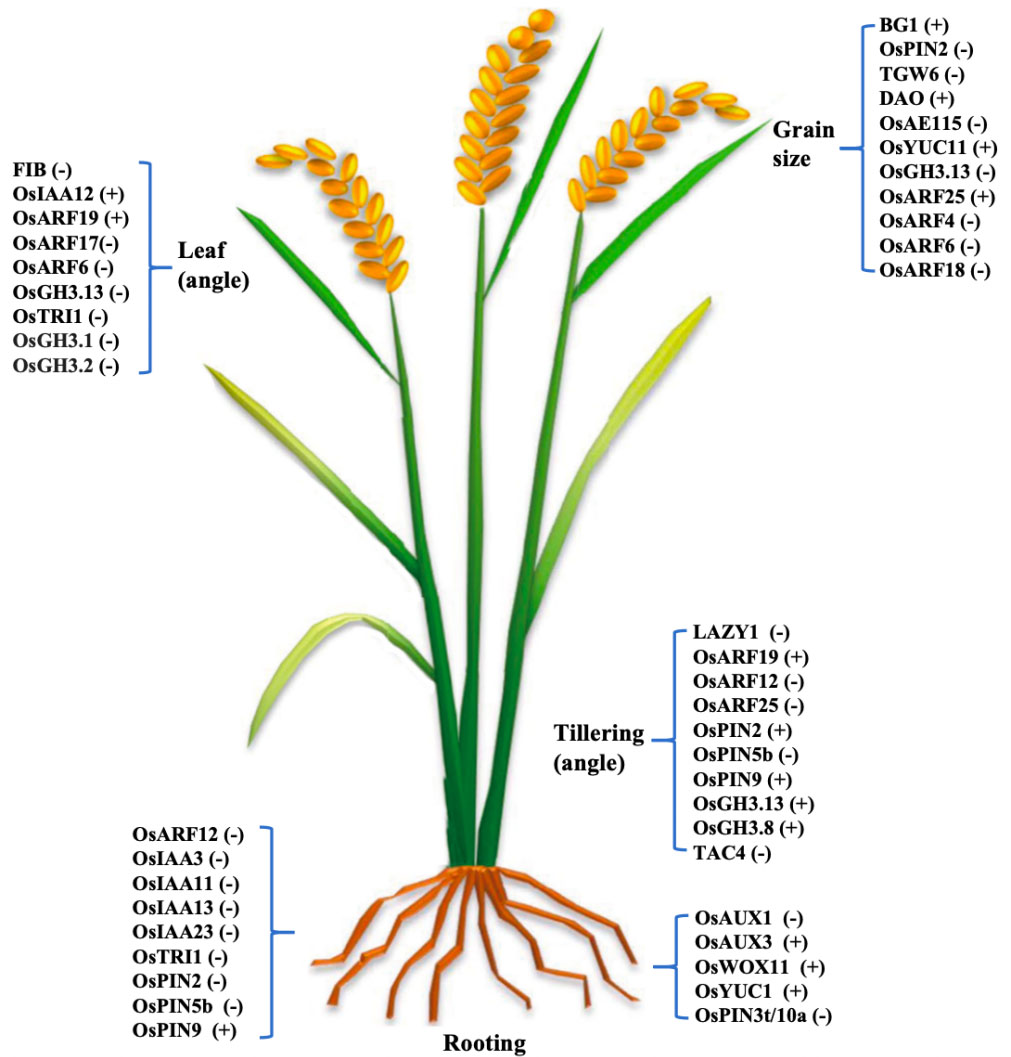
Summary of the Auxin synthesis, metabolism, transport, and signaling-related genes regulate rice root architecture (primary root, lateral root), tillering (angle), leaf (angle) development, and grain size. The plus sign (+) in brackets following a gene name indicates positive regulation, while the minus sign (-) denotes negative regulation.

OsPIN2 overexpression reduces plant height, increases tiller number and angle, and enhances auxin transport from shoot to root without altering auxin distribution in the root. It inhibits OsLazy1 expression, affecting shoot architecture (Chen et al., 2012). OsPIN2 improves aluminum tolerance by facilitating aluminum ion internalization into vacuoles and regulates root elongation and lateral root formation (Wu et al., 2014; Inahashi et al., 2018). OsPIN2 plays an important role in root gravitropic responses and determining the root system architecture in rice (Wang L. et al., 2018). High OsPIN2 expression under low phosphorus conditions modulates root growth and development (Sun et al., 2019). OsPIN9, OsPIN10a, and OsPIN10b are monocot-specific. OsPIN9, expressed in vascular tissues, is induced by ammonium but not nitrate, and is essential for ammonium-regulated tiller growth. OsPIN9 affects auxin transport between main stem and tiller bud and contributes to abiotic stress tolerance (Hou et al., 2021; Xu et al., 2022). OsPIN1b, OsPIN1c, and OsPIN9 are involved in cadmium-induced inhibition of lateral root development (Wang et al., 2021). OsPIN5b, expressed in vegetative and reproductive tissues, regulates plant architecture and yield by altering auxin homeostasis and distribution. Overexpression of OsPIN5b leads to reduced plant height, leaf number, tillers, and yield, while reduced expression increases tiller number and yield (Lu et al., 2015). Salicylic acid inhibits root growth by interfering with auxin transport through OsPIN3t and clathrin-mediated pathways (Jiang et al., 2023). OsPID, a homolog of Arabidopsis PID, regulates stigma development and cooperates with OsNPY to control spikelet and flower development. OsPID phosphorylates OsPIN1a and OsPIN1b, affecting auxin distribution and floral organ formation. It also interacts with OsMADS16 and LAX1 to regulate branching and tillering, with overexpression increasing branch number and yield per plant without affecting thousand-grain weight (Wu et al., 2020). The roles of auxin-related genes in regulating key aspects of rice development was summarized in **Figure 2**.

LA1 (LAZY1) mediates shoot gravitropism and ultimately manages tiller angle by regulating polar auxin transport. HSFA2D and LA1 regulate rice tillering angle through auxin-mediated asymmetric expression of WOX6 and WOX11 (Zhang et al., 2018a). OsBRXL4 (Brevis Radix Like 4) affects the nuclear localization of LA1 through interaction with LA1, influencing gravity response and tillering angle in rice. Overexpression of OsBRXL4 reduces LA1 nuclear localization, increasing the tillering angle, showing a semi-sporadic phenotype (Li et al., 2019a). LF1 (lateral florets 1) directly binds the promoters of OsZPR4 (LITTLE ZIPPER family gene) and OsHOX1, activating their expression and ultimately controlling rice tiller angle by regulating local auxin content (Zhang et al., 2021b). OsSPL10 (SQUAMOSA PROMOTER BINDING PROTEIN-LIKE10), affecting trichome number and length, modulates auxin sensitivity and homeostasis by regulating HL6, OsYUCCA5, and OsPIN1b, impacting epidermal hair formation and elongation (Li et al., 2021b). Auxin biosynthesis and transport are critical for coleoptile development under flooding conditions in japonica rice (Nghi et al., 2021).

## Auxin signal transduction

Auxin signals are perceived by the TIR1/AFB family of F-box proteins, which are part of the SCF (Skp1-Cullin-F-box) ubiquitin ligase complex. When auxin is present, it binds to the TIR1/AFB proteins, facilitating the interaction with AUX/IAA proteins. This interaction leads to the ubiquitination and subsequent degradation of AUX/IAA proteins, releasing the transcription factors ARFs (Auxin Response Factors), which can then activate or repress target gene expression. The TIR1/AFB-AUX/IAA-ARF module is central to auxin signaling and is conserved across plant species (Salehin et al., 2015). Members of the OsTIR1/AFB family exhibit partial redundancy in regulating key agronomic traits such as yield, tillering, plant height, root system architecture, and germination. Among these, OsTIR1, OsAFB2, and OsAFB4 mutants display more pronounced phenotypes compared to OsAFB3 and OsAFB5 mutants. Double mutants of OsTIR1 and OsAFB2 show severely compromised plant development. All five OsTIR1/AFB family members are capable of interacting with OsIAA1 and OsIAA11 proteins *in vivo*. Root elongation assays demonstrate that Ostir1/afb2-5 mutants exhibit resistance to 2,4-D treatment (Guo et al., 2021). In addition to these roles, OsTIR1 is crucial for rice grain yield and quality by modulating sugar transport into the endosperm. The expression of OsTIR1 fluctuates during the early stages of grain development and is highly expressed in the ovular vascular trace, nucellar projection, nucellar epidermis, aleurone layer cells, and endosperm. In OsTIR1 mutants, starch accumulation is reduced, leading to decreased grain yield and quality. Conversely, overexpression of OsTIR1 enhances starch accumulation, improving grain yield and quality. The transcript levels of OsARF25 are downregulated in *tir1* mutants and upregulated in OsTIR1 overexpression lines. OsARF25 can bind to the promoter of the sugar transporter *OsSWEET11* both *in vivo* and *in vitro*, and mutants of arf25 and arf25/sweet11 exhibit reduced starch content and seed size, similar to tir1 mutants (Wu et al., 2024).

OsMADS25 enhances auxin biosynthesis and transport, leading to increased auxin accumulation in roots and reduced auxin degradation, thus promoting root development. OsMADS25 directly regulates the expression of OsIAA14 by binding to the CArG-box of its promoter. The increased auxin level may reduce OsIAA14 protein accumulation, thereby modulating auxin signaling (Zhang et al., 2018b). OsIPK2 (inositol polyphosphate kinase 2) interacts directly with OsIAA11 to prevent its degradation. Co-expression of GFP-OsIPK2 (inositol polyphosphate kinase 2) delays auxin-induced degradation of Myc-OsIAA11 fusion protein in a rice protoplast transient expression system. Additionally, overexpression of OsIPK2 or its N-terminal sequence increases OsIAA11 protein accumulation in transgenic plants, leading to defects in lateral root formation and auxin response (Chen et al., 2017).

Leaf angle is a critical breeding trait that affects the photosynthesis efficiency of densely planted rice crops, influencing plant architecture and yield. Loss of function in the *Osarf6/osarf17* double mutant impairs cellulose synthesis and lignin deposition in leaf cushion cells, reducing secondary cell wall levels. This results in increased flag leaf angles and decreased grain yield under dense planting conditions. Mechanical strength measurements show a significant reduction in the leaf cushion’s ability to support the flag leaf, resembling the phenotype observed in the rice ila1 mutant (Huang et al., 2021). OsARF6 and OsARF17 can independently and cooperatively bind to the ILA1 promoter to activate its expression, with auxin-induced expression of ILA1 depending on these factors (Huang et al., 2021). Furthermore, OsARF17 plays a positive role in rice virus resistance. Viral proteins can interact directly with OsARF17, attenuating its mediated resistance. For example, the southern rice black-streaked dwarf virus P8 protein interferes with OsARF17 dimerization, disrupting its transcriptional activation, while the rice stripe virus P2 protein inhibits OsARF17’s DNA-binding ability. The Oryza mosaic virus M protein also inhibits OsARF17 transcriptional activation (Zhang et al., 2020). OsARF12 is a key mediator in the miR167-regulated grain filling process in rice. Overexpression of OsARF12 results in phenotypes that mimic the grain weight and filling traits observed in STTM/MIM167 plants. OsARF12 activates OsCDKF;2 by binding to its promoter, positively influencing grain filling and size. RNA sequencing reveals that the miR167-OsARF12 module affects cell development and hormone pathways. OsARF12-overexpressing plants and *cdkf;2* mutants exhibit altered sensitivity to auxin and brassinosteroid (BR), indicating that OsCDKF;2 mediates auxin and BR signaling (Zhao et al., 2023). A rice thermo-sensitive mutant with defects in floret development under high temperatures carries a mutation in SUPPRESSOR OF GENE SILENCING 3a (OsSGS3a). OsSGS3a interacts with OsSGS3b to modulate tasiRNA biogenesis, targeting AUXIN RESPONSE FACTORS (ARFs). OsSGS3a/b positively regulates thermotolerance, while OsARF3a/b and OsARF3la/lb negatively regulate it. Moreover, OsSGS3a negatively affects disease resistance to *Xanthomonas oryzae* and *Magnaporthe oryzae*, whereas OsARF3a/b and OsARF3la/lb positively regulate these traits (Gu et al., 2023).

The tiller angle in rice, defined as the angle between the outermost tiller and the vertical axis, is a critical trait that influences planting density and yield per unit area. OsARF12, OsARF17, and OsARF25 function redundantly in auxin response, affecting polar auxin transport and participating in the HSFA2D- and LAZY1-dependent auxin asymmetric distribution pathways, thereby controlling rice tillering angle (Li et al., 2020). As a target gene of Osa-miR167d, the expression of OsARF12 is significantly downregulated in Osa-miR167d overexpression plants and upregulated in MIM167d overexpression plants. Homozygous *osarf12* mutants exhibit an inability to detect hydrogen peroxide at infection sites, making them more susceptible to rice blast. Thus, Osa-miR167d likely regulates rice blast resistance by modulating OsARF12 expression (Zhao et al., 2020). Moreover, the OsIAA10-OsARF12-OsWRKY13 signaling pathway plays an essential role in rice antiviral defense. OsIAA10 interacts with OsARF12 to inhibit the transcription of downstream defense genes such as OsWRKY13. Exogenous auxin promotes the degradation of OsIAA10, alleviating the inhibition of OsARF12 by OsIAA10 (Qin et al., 2020). Additionally, OsARF11 negatively regulates virus resistance in rice (Qin et al., 2020). Auxin signaling module OsSK41-OsIAA10-OsARF regulates grain yield traits in rice (Ma et al., 2023). The DS1 protein interacts with OsARF11 to co-regulate the expression of D61/OsBRI1, modulating rice plant architecture through brassinosteroid signaling (Liu et al., 2018). Furthermore, OsARF4 is involved in regulating grain size and weight, as qTGW3 encodes a GSK3/SHAGGY-Like Kinase (OsGSK5/OsSK41) that interacts with and phosphorylates OsARF4 to negatively regulate these traits in rice (Hu et al., 2018). The salt tolerant mutant, *RST1*, exhibits enhanced salinity resistance and improved grain yield. *RST1* encodes an auxin response factor (*OsARF18*). Molecular analyses indicate that RST1 directly inhibits the expression of the asparagine synthetase 1 (*OsAS1*) gene. The loss of *RST1* function increases *OsAS1* expression, thereby enhancing nitrogen (N) utilization efficiency by promoting asparagine production and preventing excessive ammonium (NH^4+^) accumulation. RST1 is currently undergoing directional selection during domestication. The superior haplotype RST1 Hap III reduces its transcriptional repression activity and contributes to increased salinity tolerance and grain weight. This reveals a synergistic regulatory mechanism related to nitrogen metabolism and provides new strategies for developing salt-tolerant varieties (Deng et al., 2022). Auxin related genes involved in rice development are summarized in **Table 1**.

**Table 1:** Auxin related genes involved in rice development.

Genes	Location	Function	References
*OsPIN1/1b*	PM	Knockdown of *OsPIN1/1b* decrease the number of crown root and increase tiller number	Xu et al., 2005; Sun et al., 2018
*OsPIN2*	PM	Increased tiller number and tiller angle, improves aluminum tolerance, root gravitropic responses	Chen et al., 2012; Wu et al., 2014; Inahashi et al., 2018; Wang L. et al., 2018
*OsPIN3t/10a*	PM	Overexpression of *OsPIN3t/10a* promote crown root growth and drought tolerant	Zhang et al., 2012
*OsPIN5b*	ER	Negatively regulate plant height, tiller number, biomass, seed setting and yield	Lu et al., 2015
*OsPIN9*	PM	Response to ammonium, positively regulate tiller number	Hou et al., 2021
*OsYUCCA6*	Unknown	Regulation of lateral organs polarity development	Zhang et al., 2021a
*OsYUCCA4*	Unknown	Anther development	Song et al., 2018
*OsYUCCA1*	Unknown	Regulation IAA synthesis, crown root development	Zhang et al., 2018
*OsAUX1*	PM	Regulate primary root length, initial of lateral root and root hair development	Yu et al., 2015; Zhao et al., 2015; Giri et al., 2018
*OsAUX3*	PM	Regulate primary root elongation, initial of lateral root, root hair development, grain length and width	Wang et al., 2019; Qiao et al., 2021
*OsAUX4*	PM	Response to Pi starvation, regulate root hair development	Ye et al., 2021
*OsABCB14*	PM	Involved in auxin transport and ion homeostasis	Xu et al., 2014
*OsWOX11*	Nucleus	Crown root and shoot apical development	Zhang et al., 2018; Zhou et al., 2017
*OsARF12*	Nucleus	Knockout of *OsARF12* lead to decrease the primary root length and lower ion content, increase tiller angle, grain filling and size	Qi et al., 2012; Zhao et al., 2023
*OsARF25*	Nucleus	Knockout of *OsARF25* lead to decrease the plant height and increase the tiller angle	Li et al., 2020
*OsARF17*	Nucleus	Overexpression of *OsARF17* increase the tolerance to double-stranded RNA viruses, knockout of *OsARF17* enlarged leaf inclination and increase tiller angle, grain filling	Zhang et al., 2020; Huang et al., 2021; Chen et al., 2024;
*OsARF19/7a*	Nucleus	Overexpression of *OsARF19* led to increase leaf angle, knockout of *OsARF19* elongated basal internodes and leaves, enlarged and degenerated palea, and an additional lemma	Zhang et al., 2015; Zhang et al., 2016
*OsARF12*	Nucleus	Osa-miR167d regulate rice blast resistance by regulating the expression of *OsARF12*; *OsIAA10-OsARF12-OsWRKY13* mediated signal pathway in antiviral defense and virus anti defense	Zhao et al., 2020; Qin et al., 2020
*OsARF11*	Unknow	Negative regulation resistance to viruses, regulate plant architecture	Liu et al., 2018; Qin et al., 2020
*OsGRF7*	Nucleus	Overexpression of *OsGRF7* causes a semidwarf and compact plant architecture	Chen et al., 2020b
*OsHOX1*	Nucleus	Repress the expression of multiple *OsYUCCAs* and reduce auxin synthesis	Hu et al., 2020
*OsHOX28*	Nucleus	Repress the expression of multiple *OsYUCCAs* and reduce auxin synthesis	Hu et al., 2020
*OsGH3.8/OsMGH3*	Unknow	Modulation of plant architecture	Dai et al., 2018
OsNPR1	Nucleus/cytoplasm	Disturbance of auxin pathway, partially through indirect up regulation of OsGH3.8 expression to affects rice development	Li et al., 2016b
OsSNB/OsAE115	Nucleus	affecting grain size and weight, Rice Seed Shattering and Seed Size	Ma et al., 2019; Jiang et al., 2019
OsIAGT1/OsIAGLU	Cytoplasm	Regulate auxin homeostasis	Liu et al., 2019
*OsIAA20*	Nucleus	Reduced the sensitivity to ABA	Zhang et al., 2021d
*OsIAA12*	Nucleus	OsIAA12 can directly interact with OsARF17 to regulate leaf inclination	Chen et al., 2018
*OsIAA11*	Unknow	lateral root formation and auxin response	Chen et al., 2017
*OsIAA10*	Unknow	Knockdown of *OsIAA10* enhances the resistance to RDV infection; OsSK41-OsIAA10-OsARF regulates grain yield traits	Jin et al., 2016; Qin et al., 2020; Ma et al., 2023
*OsIAA6*	Unknow	OsIAA6 trigger auxin mediated drought response by regulating auxin synthesis genes	Jung et al., 2015
*OsTIR1*	Unknow	Regulate flag leaf inclination, primary and crown root growth, involved in nitrogen promoted tillering, seed filling	Guo et al., 2021; Wu et al., 2024
*LAZY1*	Nucleus/PM	Regulate the polar transport of auxin, affect tillering angle	Zhang et al., 2018a Li et al., 2019a
*OsRAA1*	Unknow	accumulation potassium and tolerance to moisture stress	Chen et al., 2018
*TAC4*	Nucleus	Control tiller angle	Li et al., 2021a
*TGW6*	Unknow	Hydrolyze IAA-glucose to free IAA and glucose	Ishimaru et al., 2013

The mutant *big grain1-D* (Bg1-D) shows increased grain weight, length, and width. Bg1 is a major auxin response gene, regulating cell division, elongation, and organ size (Liu et al., 2015). Tiller angle controller 4 (TAC4) regulates tiller angle by increasing IAA content and influencing auxin distribution (Li et al., 2021a). Small auxin-up RNAs (SAURs), such as OsSAUR45, are induced by IAA and mediate rice development by affecting auxin synthesis and transport (Xu et al., 2017). LC3 (leaf inclination3), a transcription inhibitor that does not bind DNA directly, interacts with LIP1 to form a heterodimer. This complex inhibits the transcription of downstream targets such as OsIAA12 and OsGH3.2, affecting auxin homeostasis and signal transduction. Consequently, LC3 regulates normal leaf development and leaf inclination (Chen et al., 2018).

Overexpression of the *OsIAA6* enhances drought resistance, whereas the knockout of *OsIAA6* leads to abnormal tiller growth (Jung et al., 2015). OsIAA20 regulates the expression of the abscisic acid response gene *OsRab21* and exhibits a positive response to both drought and salt stress (Zhang et al., 2022). OsIAA29 modulates seed development under high temperatures by competing with OsIAA21 for binding to OsARF17, thereby mediating the auxin signaling pathway in rice (Chen et al., 2024). miR408-5p typically inhibits the translation of the IAA30 protein, but in a high auxin environment, it promotes the decay of overproduced IAA30 mRNA. IPA1 (IDEAL PLANT ARCHITECTURE1), regulated by miR156, modulates leaf inclination by associating with the miR408-5p precursor promoter. The miR156-IPA1-miR408-5p-IAA30 module can also be regulated by miR393, which silences auxin receptors (Rong et al., 2024). OsIAA29 regulates seed development under high temperature by competing with OsIAA21 for binding to OsARF17, thereby modulating auxin signaling pathways in rice (Chen et al., 2024).

Auxin signaling is essential for enhancing lateral root (LR) branching under asymmetric stress conditions induced by heavy metals or NaCl. This signaling pathway operates downstream of reactive oxygen species (ROS) signaling in response to such stress, providing a mechanism for ROS to bypass transporter-mediated auxin transport and promote LR development. For instance, root cadmium (Cd) avoidance remains unaffected in the *osaux1* mutant, while the *osiaa11* mutant fails to produce any lateral roots on the cadmium side, despite the presence of ROS signals generated by asymmetric cadmium stress. These observations suggest an RBOH-ROS-auxin signaling cascade that enables rice roots to effectively respond to localized stress from heavy metals and NaCl, facilitating the remodeling of root system architecture (Wang et al., 2023). The RR25/26-CKX3-cytokinin signaling module mediates root responses to external ammonium by regulating auxin signaling in the root meristem and lateral root primordia (Li et al., 2024). Repression of the auxin receptor TIR1 by a mutant overexpressing miR393 increased rice susceptibility to RBSDV, as did mutants overexpressing the auxin signaling repressors OsIAA20 and OsIAA31. Additionally, auxin signaling mutants showed suppressed induction of jasmonic acid (JA) pathway genes in response to RBSDV, indicating that activation of the JA pathway may be involved in auxin signaling-mediated defense against RBSDV in rice (Zhang et al., 2019).

## Auxin conjugation and degradation

Auxins can form conjugates with sugars, amino acids, or peptides. These conjugated forms (e.g., IAA-glucose, IAA-aspartate) serve as inactive storage forms or are involved in transport. The conjugation is reversible, allowing for the release of free active IAA when needed (Casanova-Sáez et al., 2021). Auxin conjugation primarily includes the binding of indole-3-acetic acid (IAA) with amino acids, sugars, and alcohols, forming amide or ester bonds. Most conjugates are hydrolyzed to release free IAA, indicating that IAA conjugation may be an initial step in auxin degradation. The primary degradation pathway involves IAA oxidation, catalyzed by dioxygenase for auxin oxidation (DAO), converting IAA to 2-oxoindole-3-acetic acid (oxIAA) (Zhao et al., 2013) (**Figure 1**). OsGH3.2 (GRETCHEN HAGEN 3.2) encodes an enzyme that catalyzes IAA binding to amino acids, which was expressed asymbiotically in young rice lateral roots, influences root architecture. Its mutation leads to a “shallow” root phenotype. OsGH3.2 likely promotes arbuscular mycorrhizal (AM) fungal colonization, as mutants exhibit higher colonization levels and arbuscule incidence. In symbiosis, OsGH3.2 is expressed in cortical cells with mature arbuscules. At later stages, *Osgh3.2* mutants show elongated cortical cells and larger arbuscules compared to wild-type, indicating elevated auxin levels (Liu et al., 2022). OsGH3.2 regulates the storability of rice seeds through the accumulation of abscisic acid (ABA) and protective substances, with storability largely determining seed viability during storage. Compared to the wild type, overexpression of OsGH3.2 results in reduced IAA and ABA levels and decreased seed storability, while knockout or knockdown of this gene exhibits the opposite effect (Yuan et al., 2021).

OsNAC2 integrates auxin and cytokinin signaling to regulate root development, directly up-regulating IAA metabolism-related genes OsGH3.6 and OsGH3.8, while down-regulating the IAA signaling-related gene OsARF25 and CK oxidation gene OsCKX4. This interaction inhibits auxin activity and response, increases cytokinin content, and downregulates cyclin-dependent protein kinase genes (OsCDKs) and crown root-related genes (OsCRLs), further inhibiting root development (Mao et al., 2020). TGW6 (*THOUSAND-GRAIN WEIGHT 6*) encodes an indole-3-acetic acid-glucose hydrolase. The Nipponbare tgw6 allele affects the transition from syncytium to cytoplasm by regulating IAA supply, impacting cell number and grain length. In contrast, the loss of the *tgw6* allele in Kasalath increases rice yield through pleiotropic effects on source organs, enhancing grain weight (Ishimaru et al., 2013). The *miR156f-OsSPL7-OsGH3.8* pathway also regulates tiller number and plant height (Dai et al., 2018). Increased auxin levels in rice Dao mutants result in higher sucrose accumulation in leaves but reduced sucrose in reproductive organs. RNA-seq analysis reveals up-regulation of OsARF18 and down-regulation of OsARF2 in the lodicules of dao mutants. Overexpression of OsARF18 or knockout of OsARF2 replicates the dao mutant phenotype. OsARF2 binds to the sugar-response element (SuRE) in the OsSUT1 promoter to regulate OsSUT1 (sucrose transporter) expression, while OsARF18 represses OsARF2 and OsSUT1 expression by binding to AuxRE or SuRE. Overexpression of OsSUT1 in *dao* and *Osarf2* mutants alleviates spikelet opening and seed-setting defects (Zhao et al., 2022).

## Future perspectives

As our understanding of auxin’s role in rice growth and development evolves, several key areas warrant further exploration to maximize the potential of auxin-related genes for enhancing rice productivity and resilience. This review summarizes recent findings in auxin research and outlines future directions for effective crop improvement.

Detailed Functional Characterization of Auxin-Related Genes: Significant progress has been made in identifying genes involved in auxin synthesis, metabolism, transport, and signaling. However, comprehensive functional characterization across various developmental stages and environmental conditions is essential. Future studies should employ advanced gene editing techniques, such as CRISPR/Cas9, in conjunction with high-throughput omics technologies. This approach will help elucidate the precise roles of these genes and their interactions with other hormonal pathways. For example, utilizing CRISPR-Cas9-mediated gene editing technology to knock out *OsARF18* significantly enhances rice resistance to glufosinate, thereby enabling the development of herbicide-resistant rice varieties (Xia et al., 2023).

Systematic Analysis of Auxin Signaling Networks: While various auxin signaling pathways have been described, a comprehensive systems-level analysis of these networks remains lacking. Future research should utilize bioinformatics and network analysis tools to construct detailed signaling maps, revealing how different pathways interact and modulate rice development under diverse conditions.

Adaptation Mechanisms to Environmental Stress: Rice faces numerous environmental stresses, including drought, salinity, and heavy metal contamination. Investigating how auxin-related genes mediate stress responses is crucial for improving resilience. Future research should focus on understanding how these genes contribute to stress adaptation, ultimately aiming to develop rice varieties with enhanced tolerance to adverse conditions.

Interactions Between Auxin and Other Hormones: Auxin interacts with other plant hormones, such as ethylene, jasmonic acid, cytokinins and gibberellins, to regulate growth and development. Future studies should further explore these hormone interactions and their regulatory mechanisms. Integrating metabolic pathway analysis with hormonal signaling studies will provide a more comprehensive understanding of how auxin coordinates with other hormones to influence rice growth.

Application of Precision Breeding Techniques: Advancements in modern biotechnological tools offer opportunities for precise gene editing and functional modifications in rice. Future research should focus on translating insights gained from auxin-related gene studies into practical breeding strategies. Developing rice varieties with optimized growth characteristics and enhanced environmental adaptability through targeted genetic improvements will be crucial for sustainable agriculture.

Looking ahead, it is imperative to integrate multidisciplinary approaches, combining functional genomics, environmental stress adaptation, hormonal interactions, and precision breeding. Collaborative efforts between researchers, breeders, and agronomists will be essential to translate laboratory findings into field applications. Additionally, exploring the role of auxin in nutrient uptake and interactions with soil microorganisms could further enhance our understanding of plant-soil dynamics, ultimately contributing to more sustainable agricultural practices.

In summary, as research techniques advance and our understanding deepens, investigating the roles of auxin-related genes in rice growth and development offers promising avenues for improving rice yield and resilience. By integrating diverse research strategies, we can provide robust solutions to the challenges of global food security and sustainable agriculture.
